# Integrated analysis identifies DUSP5 as a novel prognostic indicator for thyroid follicular carcinoma

**DOI:** 10.1111/1759-7714.13270

**Published:** 2019-12-10

**Authors:** Qian Zhang, Yiqian Xing, Shan Jiang, Chunmei Xu, Xiaojun Zhou, Rui Zhang, Tianyue Xie, Zhiwei Zou, Piyun Gong, Huangao Zhu, Dongmei Zhang, Huimei Ma, Lin Liao, Jianjun Dong

**Affiliations:** ^1^ Department of Endocrinology Qilu Hospital, Shandong University Jinan China; ^2^ Division of Endocrinology, Department of Internal Medicine, Shandong Provincial Qianfoshan Hospital The First Hospital Affiliated with Shandong First Medical University Jinan China; ^3^ Department of Endocrinology, Shandong Provincial Qianfoshan Hospital Shandong University Jinan China

**Keywords:** Differentially expressed gene, DUSP5, papillary thyroid carcinoma, prognosis, thyroid follicular carcinoma

## Abstract

**Background:**

Differentiated thyroid cancer involves thyroid follicular carcinoma (FTC) and papillary thyroid carcinoma (PTC). Patients with FTC have a worse prognosis than those with PTC for early metastasis through blood of FTC. However, the mechanism of poor prognosis of FTC is still unclear. Here, we aim to evaluate the role of DUSP5 in the prognostic evaluation of FTC.

**Method:**

We searched the Gene Expression Omnibus (GEO) database for the differentially expressed genes (DEGs) between FTC and PTC, and then combined with survival analysis of cBioPortal database to locate the gene significantly related to prognosis. Tissue microarrays and clinical tissues were used to examine DUSP5 expression by immunohistochemical (IHC) staining between FTC and PTC tissues. In vitro experiment, proliferation, migration and invasion of FTC were observed after regulation of DUSP5 by transfection of siRNA and plasmids, respectively.

**Results:**

After searching the GEO database, 26 DEGs were found. DUSP5 was significantly associated with prognosis of FTC in combination with survival analysis. Data of IHC staining showed lower expression of DUSP5 in FTC compared to PTC tissues. Furthermore, overexpression of DUSP5 inhibited the proliferation, migration and invasion accompanied with low level of MMP9 and Vimentin and high level of E‐cadherin. Nevertheless, inhibition of DUSP5 ameliorated above damaging effect on the proliferation, migration and invasion.

**Conclusion:**

DUSP5 was differentially expressed in FTC and PTC tissues. Low level of DUSP5 in FTC participates in the high frequency of metastasis, and further contributes to poor prognosis of FTC. DUSP5 could be served as a novel therapeutic target for FTC.

## Introduction

Thyroid carcinoma (TC) incidence has increased dramatically over the past decades.^1^, ^2^ The public generally ascribes this increase to improved diagnostic accuracy.^3^ Differentiated thyroid cancer (DTC) accounts for most TC and is usually subdivided into papillary thyroid carcinoma (PTC) and follicular thyroid carcinoma (FTC).^4^, ^5^ The incidence of epithelial‐derived DTC has been also steadily increasing for at least two decades, which is mainly ascribed to increasing incidence of FTC.^6^ It is unclear to what extent they are misdiagnosed as PTC due to their similar incidence and survival without metastasis of FTC.^7^, ^8^ More importantly, clinicians always regard them equally without discrimination and have ignored the differences between them, such as invasiveness and prognosis.^9^

FTC is the second most common type of invasive thyroid cancer, and accounts for approximately 10% of allTC.^10^ Furthermore, widely invasive and unspecified FTC confers the least favorable prognosis.^11^, ^12^ Patients with FTC usually suffer a higher risk of invasion and metastasis, and further aggravate the global burden of life‐threatening disease.^13^

Normally, the degree of malignancy of TC, especially for FTC, cannot be ascertained unless histological evaluation of the surgically excised thyroid tumor is performed.^14^ Thus, it is imperative to identify a potential therapeutic target to improve the poorer survival rate of FTC patients than PTC. In addition to tumor growth,^15^ tumor migration or invasion constitutes the invasiveness of thyroid malignancy. Tumor cells possess a broad spectrum of migration and invasion mechanisms to invade adjacent tissues and to travel to distant sites and escape identification, which indicates a poor prognosis.^16^, ^17^ Patients with distant metastasis of FTC were found to have a nearly seven‐fold higher mortality rate.^18^ However, the molecular mechanisms underlying the malignancy of FTC due to metastasis are still far from being completely elucidated.

Recent advances in medical genetics have significantly facilitated efforts to uncover the mechanism of human diseases. Progress in genetic testing could identify molecular abnormalities related to the pathophysiology, and therapeutic targets of various diseases.^19^ A breakthrough in next generation sequencing (NGS) and microarray analysis provide reliable approaches for evaluation of functional DNA variations in many cancers.^20^ Genome sequencing data of NGS and microarray related to cancer development and progression were acquired by searching the Gene Expression Omnibus (GEO) database to help track neoplasm advance at the genetic level. Thyroid cancer heterogeneity, the distinction of molecular subtypes of thyroid cancer and complexity caused by genetic and epigenetic aberrations can be identified by NGS and microarray analysis, which may provide further potential diagnostic and therapeutic targets.^21^

In our study, by searching the GEO database, three datasets (GSE27155, GSE53157 and GSE29315) which involved 26 FTC tissues and 67 PTC tissues were used to analyze the differentially expressed genes (DEGs) between FTC and PTC and 26 DEGs were found. In combination with the data of survival analysis by cBioPortal database, dual‐specificity phosphatase 5 (DUSP5), a member of dual‐specificity phosphatases (DUSPs) family was observed to be significantly correlated with the prognosis of TC. Moreover, in vitro experiments further confirmed that DUSP5 participated in the regulation of proliferation, migration and invasion of FTC.

## Methods

### Data collection and microarray data

We searched the GEO database (https://www.ncbi.nlm.nih.gov/geo/) for publicly available studies until 30 June 2019 using the following keywords: “Thyroid carcinoma” (study keyword), “*Homo sapiens*” (organism), “Expression profiling by array” (study type).^22^ The inclusion criteria for studies were as follows: (i) samples diagnosed with FTC tissue samples and PTC tissue samples; (ii) gene expression profiling of mRNA and (iii) sufficient information to perform the analysis. After a systematic review, three gene expression profiles (GSE27155, GSE53157 and GSE29315) were collected for analysis. The GSE27155 dataset contained 13 FTC tissue samples and 51 PTC tissue samples; the GSE53157 dataset included 4 FTC tissue samples and 7 PTC tissue samples; The GSE29315 dataset contained 9 FTC tissue samples and 9 PTC tissue samples. Platforms used for gene profiling of GSE27155, GSE53157 and GSE29315 were GPL96, GPL570, GPL8300 (Affymetrix), respectively.

### Data processing of DEGs

To identify common DEGs in the three microarray datasets, we used GEO2R (http://www.ncbi.nlm.nih.gov/geo/geo2r),^22^ an interactive web tool that compares two or more groups of samples under the same experimental conditions in a GEO dataset. The genes that satisfied the inclusion criteria of *P* < 0.05 and |logFC| ≥ 1 were identified as DEGs and included in the study.

Bioinformatics & Evolutionary Genomics, an online website tool, was used to identify common DEGs in these three datasets and to plot Venn diagrams (http://bioinformatics.psb.ugent.be/webtools/Venn/). Each circle represented a dataset, and the overlap between the circles amounted to the overlap between the datasets.

### Survival analysis of DEGs

cBioPortal (http://www.cbioportal.org/), an open platform for exploring multidimensional cancer genomics data, was used for searching survival information of TC patients including 516 samples. The relapse free and overall survival information were based on TCGA database. Overall survival (OS) was analyzed by the Cox proportional hazards regression model adjusted by sex, age, and tumor stage. The log rank *P*‐value were calculated and displayed on the plot.

### Tissue collection

A total of 10 paired human FTC tissues and PTC tissues were acquired from patients who had been diagnosed with primary FTC or PTC by pathological assessment of tissues and undergone surgeries with complete prognostic information at the Qilu Hospital of Shandong University. No local or systemic radiotherapy, or/and targeted therapy and chemotherapy were conducted. The study was approved by the Research Ethics Committee of Qilu Hospital. Informed consents were obtained from all participating patients.

### Immunohistochemical analysis

The tissue microarrays (TMAs) were purchased from US Biomax, Inc. (TH8010a Rockville, MD, USA), which included 20 FTC and 44 PTC specimens. The clinical information of the specimens was provided by the manufacturer. Human TC tissues, obtained from Qilu Hospital of Shandong University, also were verified expression of DUSP5.

Tissue paraffin sections were cut at a thickness of 4 μm. They were then deparaffinized in xylene and rehydrated through graded alcohol, and heat induced antigen retrieval used Tris‐EDTA buffer (1 mM EDTA, 10 mM Tris, 0.05% Tween 20, pH 9.0) for 20 minutes. The slides were incubated with 3% H_2_O_2_ for 10 minutes before blocking with 5% goat serum, in order to block the activity of endogenous peroxidase. The slides were incubated with dual‐specificity phosphatase 5 (DUSP5) (1:200, ab200708, Abcam, Cambridge, USA) overnight at 4°C. After washing with PBS, the slides were incubated with peroxidase enzyme‐conjugated goat anti‐rabbit secondary antibody (#PV9001, 1:400, Zhongshan, Beijing, China) for 30 minutes at 37°C. Results of immunohistochemical staining were recorded as an average positive area (yellowish‐brown color area) by using Diaminobenzidine tetrahydrochloride (ZSBIO, Beijing, China). The slides were then counterstained with hematoxylin.

### Cell culture and transfection

FTC‐133 cell line were purchased from Shanghai Zhong Qiao Xin Zhou Biotechnology Co., Ltd. Duplex plasmids and siRNA targeting human DUSP5 were designed and synthesized by GenePharma (Shanghai, China). Empty plasmids and siRNA were used as a control. FTC‐133 cells line (1 × 10^5^ cells per well) were seeded in six‐well plates (Corning‐Costar, US) and grown to 70%–90% confluence. The plasmids and siRNA transfections were carried out with Lipofectamine 2000 (Invitrogen, US) according to the manufacturer's instructions. Briefly, Lipofectamine 2000 and plasmids was diluted into Opti‐MEM medium (Gibco, US) with a 1:1 ratio, respectively. The mixture was then incubated for 20 minutes at room temperature. The mixture was added to the cells, which were incubated for 48 hours in a 5% CO_2_ atmosphere at 37°C. After the transfection, RT‐PCR and western blotting were used to examine the transfection efficacy.

### Quantitative real‐time polymerase chain reaction (qRT‐PCR)

Total RNA was extracted from cells by Trizol, respectively. RNA was treated with deoxyribonuclease to dislodge contaminating genomic DNA (Takara Bio Inc., Japan) before reverse transcription. The complementary DNA was synthesized in a 20 μl volume using the Light Cycler kit (Takara Bio Inc., Japan) according to the manufacturer's instructions. β‐actin was as an internal control for DUSP5. The relative expression level between treatments was calculated by 2^−(ΔCtsample − ΔCtcontrol)^. The primers sequences: DUSP5 F:ATCCTGAGTGTTGCGTGGATGTA R:CTCGCACTTGGATGCATGGTA

β‐actin F:TGGCACCCAGCACAATGAA R:CTAAGTCATAGTCCGCCTAGAAGCA

### Western blot analysis

Protein concentrations in cell extracts were determined by Bio‐Rad DC Protein Assay kit (Bio‐Rad Laboratories, Inc., Hercules, CA, USA). Equal amounts of protein fractions of lysates were resolved over 10% SDS‐PAGE and transferred to PVDF membrane (EMD Millipore, Billerica, MA, USA). The membranes were incubated with rabbit anti‐DUSP5 (1:1000, ab200708, Abcam, Cambridge, USA), rabbit anti‐E cadherin (1:1000, ab194982, Abcam, Cambridge, USA, rabbit anti‐Vimentin (1:1000, ab92547, Abcam, Cambridge, USA), rabbit anti‐MMP9(1:500, ab38898, Abcam, Cambridge, USA), mouse anti‐β‐actin (1:1000; A1978, Sigma‐Aldrich; Merck KGaA) overnight at 4°C. The next day, corresponding peroxidase‐labeled goat anti‐rabbit and anti‐mouse secondary antibody (1:10 000) were used as the second antibody, respectively. The peroxidase activity was detected using the FluorChem E enhanced chemiluminescent system (ProteinSimple, San Jose, CA, USA). And ImageJ software (National Institutes of Health, USA) was used to quantify the densitometer of the optical density of the bands

### Proliferation, migration and invasion assays

The effect of DUSP5 on cell proliferation was tested by the 5‐ethynyl‐2′‐deoxeuridine (EdU) assay kit (RiboBio Co., Ltd., Wuhan, China). Briefly, cells were cultured in 96‐well plates after being transfected with DUSP5 siRNA versus control and plasmids versus control for 48 hours. Cells were then incubated with 50 μm of EdU for an additional 2 hours at 37°C. Cells were fixed with 4% formaldehyde for 30 minutes, and then incubated with glycine (2 mg/ml) for 5 minutes, and treated with 0.5% Triton X‐100 for 10 minutes. After being washed with PBS, cells were incubated with 100 μl of 1 × Apollo reaction cocktail for 30 minutes and treated with 0.5% TritonX‐100. DNA was stained with Hoechst 33342 stain for 30 minutes to reveal all the nucleus and was then visualized with an Olympus FSX100 imaging system (Olympus, Tokyo, Japan). Five groups of confluent cells were randomly selected from each sample image and analysis. The assay was repeated at least three times.

The effect of DUSP5 on cell migration was tested using a 24‐well Transwell (Corning‐Costar, US) and membrane insets were coated with cells. Briefly, the cells were treated with DUSP5 siRNA versus control and plasmids versus control for 48 hours prior to the migration assays. Cells were resuspended in serum‐free culture medium, followed by transfer into the Transwell at a density of 2 × 10^4^ cells/100 ul in the upper chamber, while 10% serum culture medium was placed in the bottom of plates. After incubating for 24 hours in a 5% CO_2_ atmosphere at 37°C, migrated cells were stained with crystal violet (Beyotime Biotechnology, C0121) and then counted under an Olympus FSX100 imaging system (Olympus, Tokyo, Japan). Five groups of confluent cells were randomly selected from each sample image and analysis. The assay was repeated at least three times. For invasion assays, other experimental procedures were the same as the migration experiment, except Matrigel (BD Biosciences)‐coated Transwell chambers were used in the upper chamber for assessing cell invasiveness.

### Statistical analysis

All statistical analyses were performed using Statistical Product and Service Solutions (SPSS) 19.0 software (from IBM). A Student's t‐test was used to assess significance for data within two groups. All data are presented as the means ± SEM, and significance was set at *P* < 0.05.

## Results

### Integrated analysis of three GEO datasets identified 26 significantly DEGs in FTC and PTC

**Figure 1:**
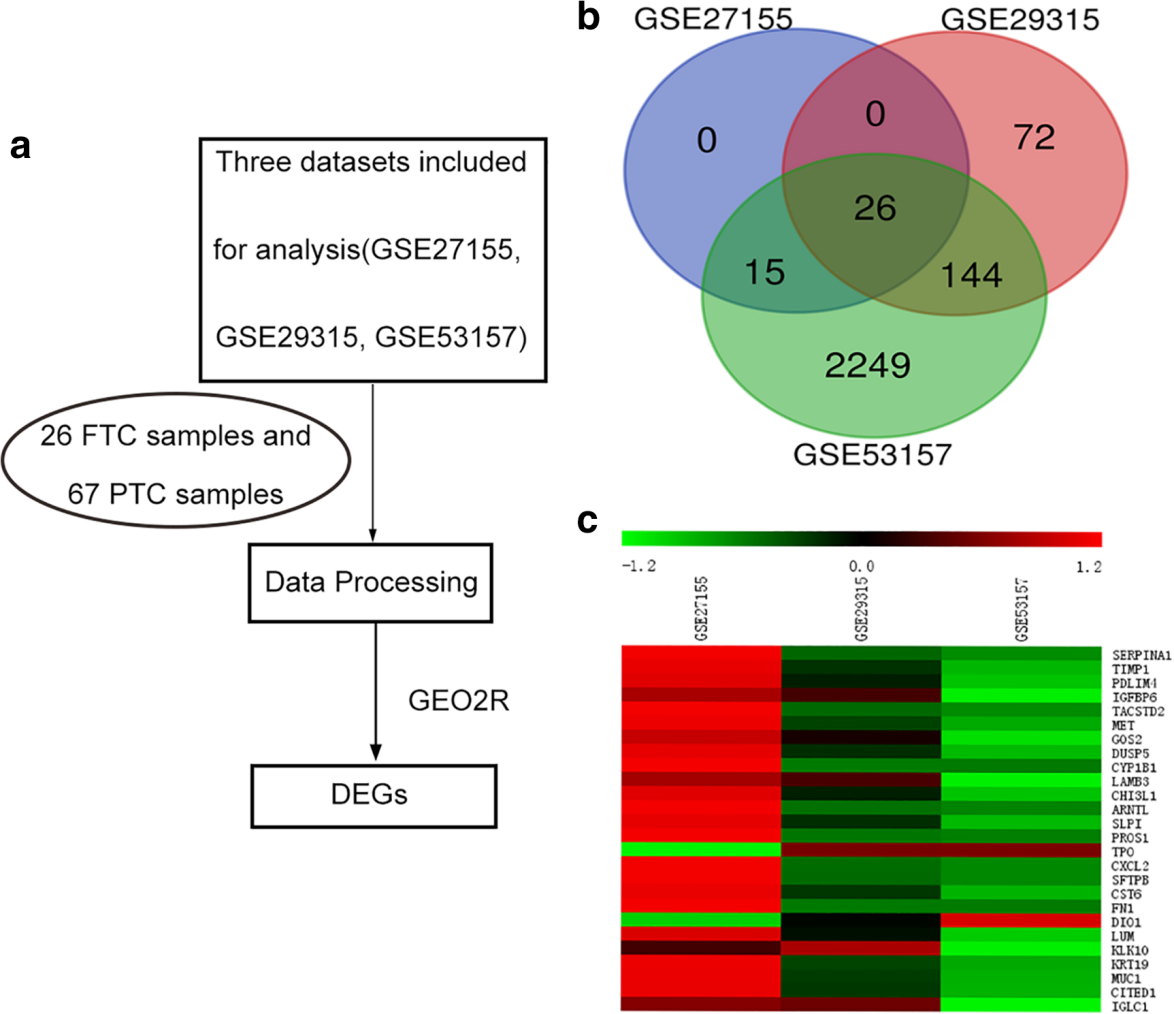
Integrated analysis of GEO datasets identified DEGs in FTC and PTC. (**a**) Flow chart of the analysis performed in the present study. GSE27155, GSE29315, GSE53157 as downloaded in order to identify DEGs between FTC and PTC. (**b**) Venn diagrams of common DGEs combined with three datasets (GSE27155, GSE29315, GSE53157). Each circle represents a dataset, and the overlap between the circles amounts to the overlap between the datasets. (**c**) Heat map of the DEGs (2 upregulated genes and 24 downregulated genes). Red, relatively upregulation; green, relatively downregulation (after correction).

After searched the GEO datasets, we identified three datasets (GSE27155, GSE29315, GSE53157) that included FTC and PTC samples (26 FTC samples and 67 PTC samples). The study selection flow chart for this integrated analysis is shown in Figure 1a.

The GEO2R online analysis tool was applied to detect the DEGs, using adjust *P‐*value < 0.05 and |logFC| ≥1 as cutoff criteria. To identify at a glance genes that were up‐ or downregulated in FTC patients, PTC patients, or both, we found the common DEGs as shown in Venn (Fig 1b). Of these, 26 gene expressions were commonly differential (Table 1), including 2 upregulated genes and 24 downregulated genes in FTC tissue samples compared with PTC tissue samples. The heat map is shown in Figure 1c.

**Table 1:** Twenty‐six differently expression genes discriminate FTC from PTC

	GSE27155		GSE29315		GSE53157		
Gene	Log2FC	*P*‐value	Log2FC	*P*‐value	Log2FC	*P*‐value	Up or down
SERPINA1	−1	1.22E−11	−3.47	2.77E−06	−4.52	3.66E−12	Down
TIMP1	−1.18	2.32E−21	−2.30	8.92E−06	−3.46	1.07E−08	Down
PDLIM4	−6.11E−01	1.54E−15	−1.74	5.95E−06	−3.89	3.24E−07	Down
IGFBP6	−1.41	1.12E−20	−1.78	3.00E−04	−4.89	4.02E−07	Down
CXCL2	−1.19	3.32E−12	−2.94	3.30E−07	−3.25	2.67E−04	Down
TACSTD2	−1.01	3.51E−07	−4.18	1.82E−02	−6.64	7.02E−07	Down
MET	−2.08	4.38E−02	−1.58	3.06E−04	−3.25	1.51E−06	Down
G0S2	−1.02	2.65E−12	3.06E−04	4.76E−06	−4.66	1.59E−06	Down
DUSP5	−1.02	2.61E−17	−2.16	1.19E−07	−3.524	1.89E−06	Down
CYP1B1	−1.11	7.57E−15	−2.33	1.02E−04	−2.33	2.17E−06	Down
LUM	−1.17	4.41E−11	−2.23	2.30E−04	−5.25	7.16E−04	Down
LAMB3	−1.04	2.22E−23	−1.35	3.87E−05	−3.76	3.49E−06	Down
CHI3L1	−1.83	3.35E−18	−3.09	3.20E−04	−6.55	1.01E−05	Down
ARNTL	−7.35E−01	1.08E−10	−2.31	4.39E−08	−2.47	1.59E−05	Down
SLPI	−1.03	2.29E−13	−2.29	8.78E−05	−4.03	4.27E−05	Down
PROS1	−1.09	6.48E−22	−2.71	8.14E−06	−2.79	5.41E−05	Down
FN1	−7.24E−01	4.57E−20	−2.76	2.07E−05	−2.79	4.18E−04	Down
SFTPB	−1.24	1.10E−09	−3.77	2.39E−05	−4.57	3.41E−04	Down
CST6	−1.03	9.32E−12	−2.54	6.85E−07	−4.77	3.76E−04	Down
KLK10	−1.48	1.97E−10	−1.14	4.11E−04	−3.73	1.43E−03	Down
KRT19	−1.13	1.09E−09	−2.70	2.38E−06	−4.22	2.27E−03	Down
MUC1	−4.73E−01	4.59E−07	−1.44	2.61E−04	−2.13	2.87E−02	Down
CITED1	−1.32	1.99E−08	−2.34	6.04E−04	−3.21	5.91E−03	Down
IGLC1	1.99E−08	1.67E−03	−0.95	1.84E−02	−2.69	2.98E−02	Down
TPO	1.17	1.56E−07	4.164	2.31E−07	4.18	1.22E−04	Up
DIO1	1.15	2.05E−08	3.76	1.88E−05	4.69	6.16E−04	Up

**Figure 2:**
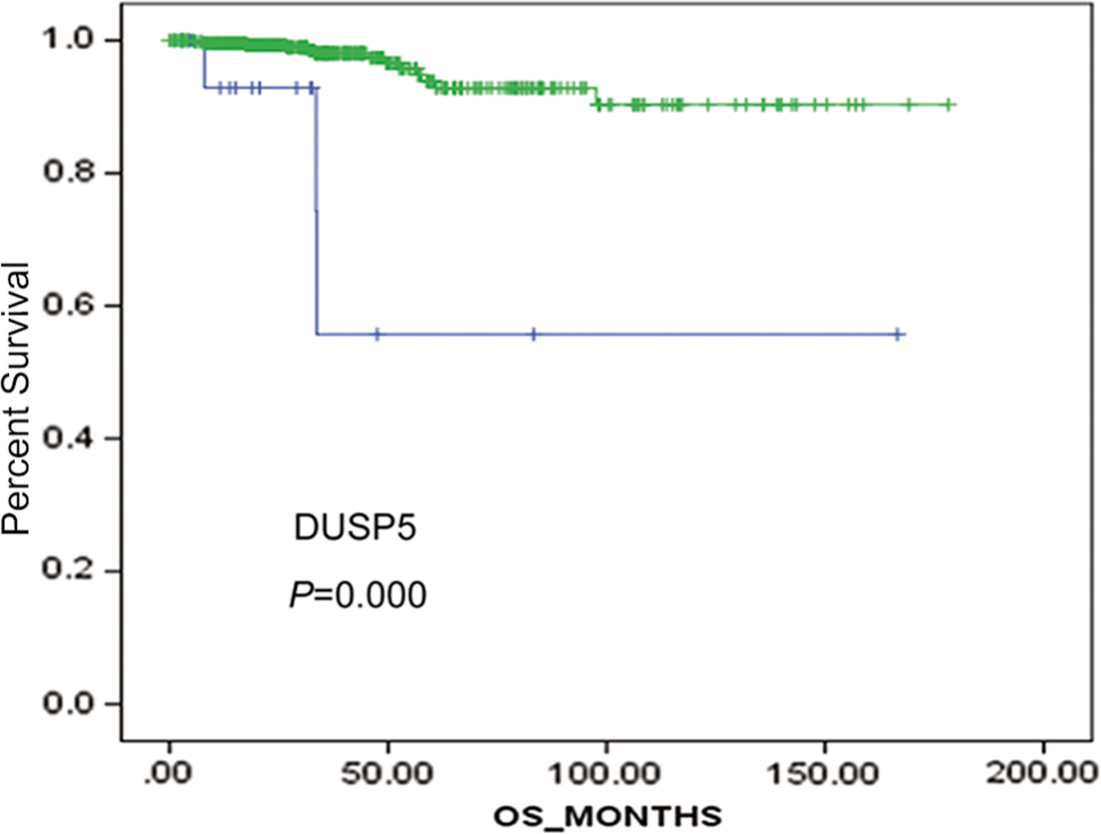
Kaplan‐Meier survival analysis was conducted to determine the associations between the DUSP5 expression levels and survival prognosis in cBioPortal. cBioPortal, an platform for exploring multidimensional cancer genomics data; DUSP5, Dual‐specificity phosphatase 5. (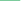) High expression and (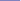) low expression.

DEGs expression data were available for all patients from the cBioPortal online website. The correlations between each gene and OS were analyzed. The results indicated that the expression level of DUSP5 (*P <* 0.05) was significantly correlated with OS (Fig 2). Another 24 DEGs expression level was not significantly associated with OS (Fig S1). The DEGs of IGLC1 was unrecorded in the database. Furthermore, a joint effects analysis of DUSP5 with OS was performed, which demonstrated that patients with low expression levels of DUSP5 had a worse OS compared with those with high expression levels of DUSP5.

### Expression of DUSP5 in TC samples

**Figure 3:**
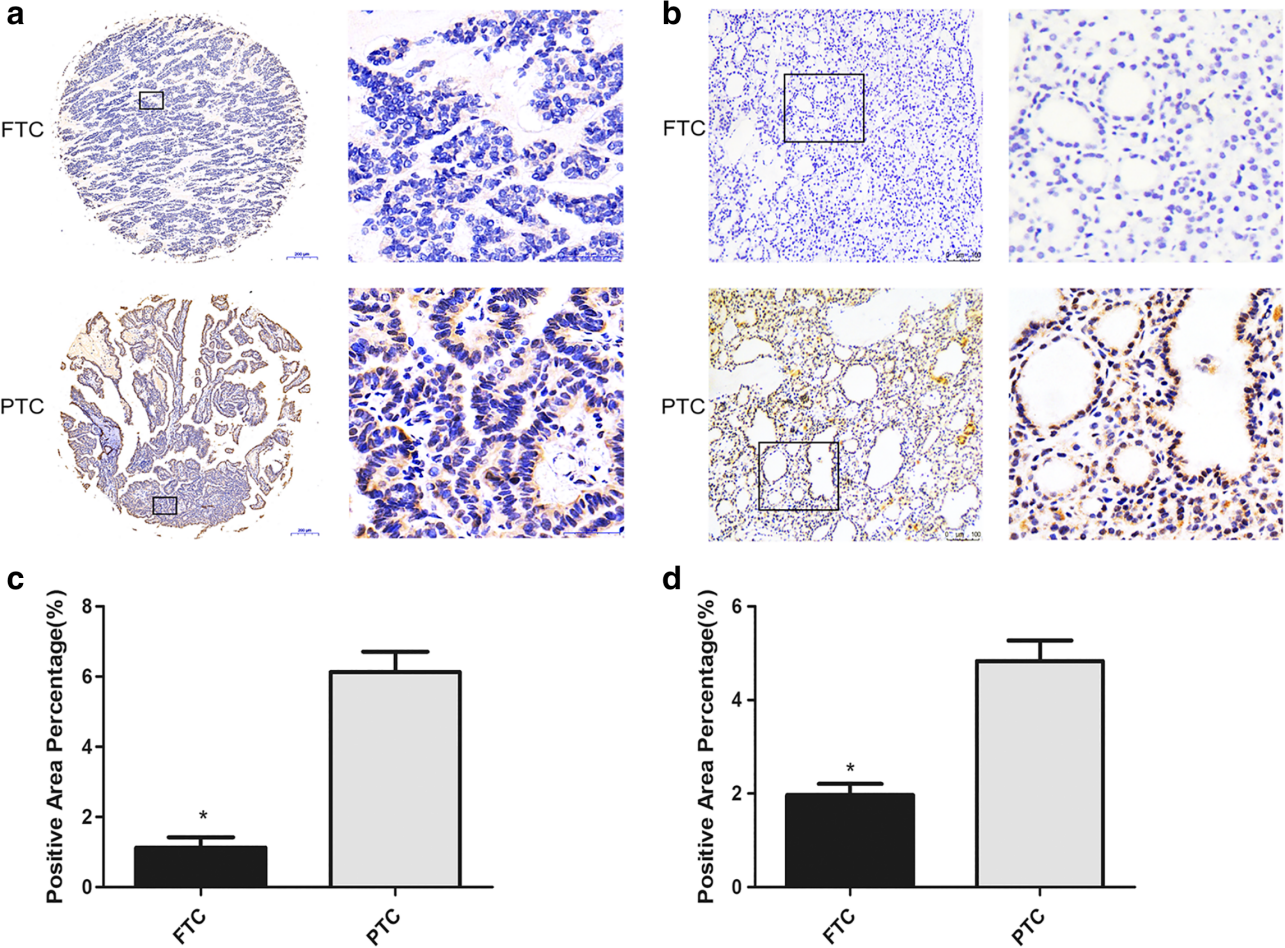
IHC staining for DUSP5 in tissues. (**a**) DUSP5 expression in TMAs of FTC and PTC samples. Representative images of DUSP5 immunohistochemistry on FTC tissues and PTC tissues. Magnification, x50 (left panels) and x400 (right panels). (**b**) DUSP5 expression in clinical samples of FTC and PTC. Representative images of DUSP5 immunohistochemistry on FTC tissues and PTC tissues. Magnification, x100 (left panels) and x200 (right panels). (**c**) Quantification of positive‐staining for DUSP5 in TMAs of FTC and PTC groups. DUSP5 was significantly less in FTC than PTC samples. The values denote the positive area/ total area ± SEM. *, statistically significant difference (*P* < 0.05). (**d**) Quantification of positive‐staining for DUSP5 in clinical samples of FTC and PTC groups. DUSP5 was dramatically less in FTC than PTC samples. The values denote the positive area/total area ± SEM. *, statistically significant difference (*P* < 0.05).

We subsequently performed an immunohistochemical (IHC) analysis of DUSP5 expression via a tissue microarray which included 20 FTC samples and 44 PTC samples. IHC staining of TMAs showed clear and distinguishable cytoplasm staining for DUSP5 in tumor tissues, and DUSP5 expression was significantly lower in FTC tissues compared to PTC tissues (*P* < 0.05, Fig 3a).

Furthermore, we determined DUSP5 protein expression in clinical samples using IHC analysis in samples of FTC and PTC (Fig 3b). According to the staining location, the analysis of DUSP5 protein expression was shown in Figure 3c,d. The expression level of DUSP5 in FTC tissues was significantly lower than the expression of PTC (*P* < 0.05). These data demonstrated that the downregulation of DUSP5 might play critical roles in FTC development and progression.

### Silence of DUSP5 expression promoted FTC‐133 cells proliferation, migration, invasion

**Figure 4:**
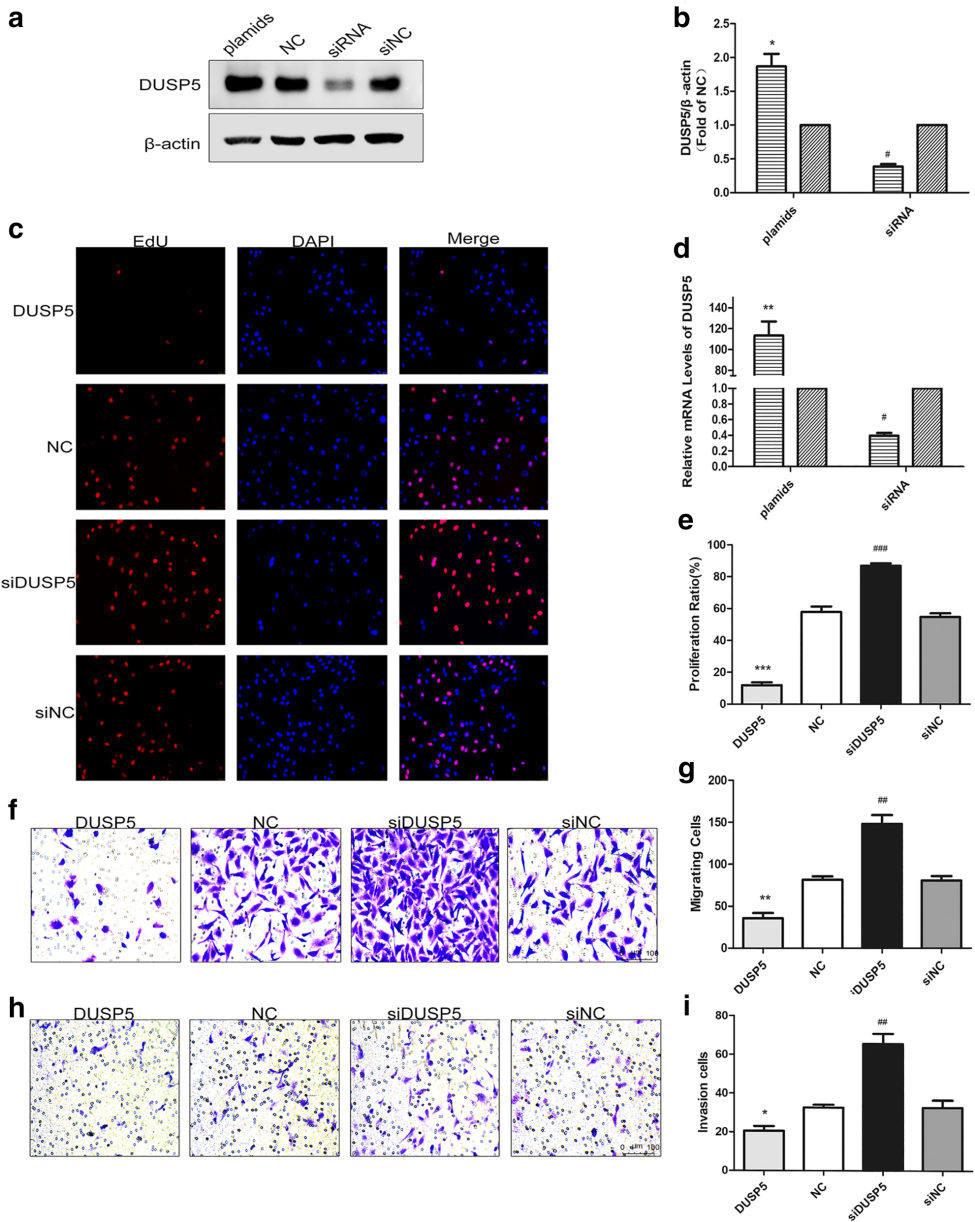
The regulation of DUSP5 in FTC‐133 cell line and the proliferation, migration and invasion of FTC‐133 cell line after regulation of DUSP5. (**a, b**) Western blot and analysis of DUSP5 protein expression after regulated by plasmids and siRNA of DUSP5 and empty vectors, respectively. Data are expressed as mean ± SEM. *^#^, statistically significant difference (*P* < 0.05). (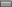) DUSP5 and (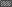) NC. (**c**, **e**) EdU assay showed that increased EdU‐positive cells were observed with down‐regulation of DUSP5 in FTC‐133. (**e**) Rate of EdU‐positive cells. *^#^, statistically significant difference (*P* < 0.05). (**d**) DUSP5 mRNA expression was detected by real‐time PCR after transfected by DUSP5 siRNA, plasmids and empty vectors, respectively. *^#^, statistically significant difference (*P* < 0.05). (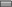) DUSP5 and (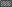) NC. (**f**, **g**) Transwell migration assay of FTC‐133 cells after upregulating and intervening DUSP5 expression. The purple cells were stained with crystal violet and presented as migrated cells. The images of transwell migration assay were taken at ×200 magnification and scale bar was 75 μm. The quantitative data of the migration of FTC‐133 cells. *^#^, statistically significant difference (*P* < 0.05). (**h**, **i**) Matrigel (BD Biosciences)‐coated Transwell invasion assay of FTC‐133 cells after regulated by plasmids and siRNA of DUSP5 and empty vectors. The purple cells were stained with crystal violet and presented as invasion cells. The images of transwell invasion assay were taken at ×200 magnification and scale bar was 75 μm. The quantitative data of the invasion of FTC‐133 cells. *^#^, statistically significant difference (*P* < 0.05). FTC‐133, thyroid follicular cancer cells

**Figure 5:**
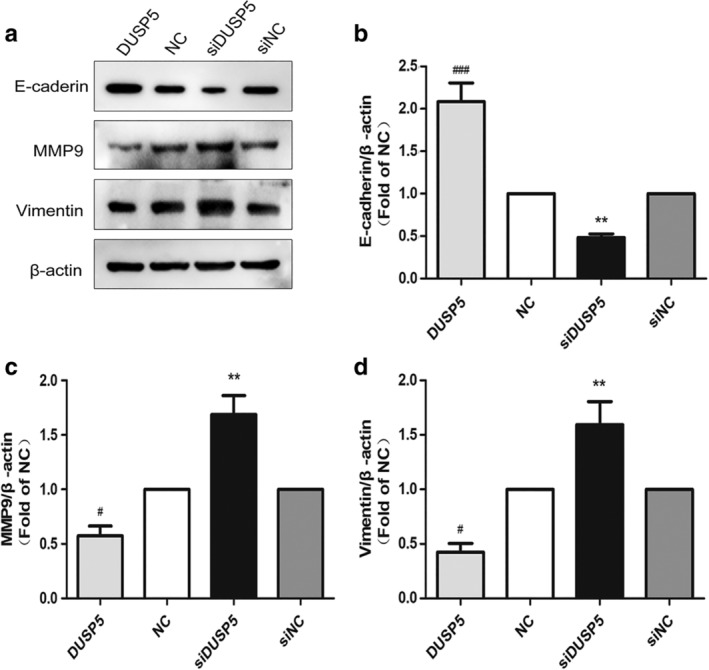
Migration and invasion relative protein expression level in FTC‐133 cell line after regulated. (**a**–**d**) Western blot confirmed the change of E‐cadherin, Vimentin and MMP9 protein levels after transfected plasmids and siRNA of DUSP5. Data are expressed as mean ± SEM. *^#^, statistically significant difference (*P* < 0.05).

We further investigated the effects of DUSP5 on biological functions in FTC. DUSP5 plasmids were transfected into FTC‐133 cells to construct a DUSP5 overexpression model, and DUSP5 siRNA were transfected into FTC‐133 cells to establish a DUSP5 low‐expression model (Fig 4a,b,d). On this basis, a EdU assay was conducted to detect the cell proliferation. The results indicated that FTC‐133 cells with knockdown DUSP5 grew faster than the control group, while cells with overexpressed DUSP5 had a lower rate of proliferation (Fig 4c,e). Additionally, knockdown of DUSP5 group of FTC‐133 cells had a greater number of cell migration than the control group (Fig 4f,g). In comparison, the number of migrated cells with an overexpression of DUSP5 was less than in the control group (Fig 4f,g). Similarly, the results of invasion also showed that DUSP5 knockdown dramatically induced cell invasion, while DUSP5 overexpression led to the inhibition of cell invasion (Fig 4h,i). The changes about migration and invasion related protein in FTC‐133 cells were shown in Figure 5a–d. Compared with the control, upregulation of DUSP5 inhibited the proliferation and migration of FTC‐133 accompanied with low level of MMP9 and Vimentin and high level of E‐cadherin, and this effect could be reversed by the DUSP5 silence.

## Discussion

The treatment of DTC is generally involved in total or subtotal thyroidectomy followed by radioactive iodine therapy, and then the patients receive long‐term thyroid‐stimulating hormone suppression.^4^, ^23^ A majority of patients with DTC have an excellent prognosis, whereas, a few patients with extensive invasion and distant metastasis frequently do not respond to treatments and experience worse prognosis.^24^ Compared with PTC, patients with FTC are prone to distant metastasis due to its transfer way through blood.^25^ Currently, many clinicians confuse them for their common well‐differentiated stage but ignore their difference in the way of metastasis which severely affects the prognosis and survival of patients. Understanding the specific mechanisms involved in FTC invasion and metastasis is critical in order to achieve early detection of the distant metastasis and develop new treatments specifically targeted for these patients.

The goals of the current study were to identify the candidate driver gene in FTC through analysis of multiple datasets of genes expression arrays and to validate their biological functions and significance for clinical prognosis. The GEO database at the US National Center for Biotechnology Information (NCBI) has emerged as one of the major public repositories for gene expression data, especially for DEGs.^22^, ^26^, ^27^ According to the GEO database, we conducted a comprehensive literature search to identify microarray‐based gene expression profiling studies, and attempted to combine the datasets of these studies to find a DEG spectrum to distinguish FTC tissues and PTC tissues. Combined with the purpose of our research, we finally found that low expression of DUSP5 was correlated with the poor prognosis of TC by bioinformatics and survival analysis. With a human tissue microarray and patient tissues with FTC or PTC, we then confirmed the low expression of DUSP5 in FTC. Moreover, the mechanism of DUSP5 regulation on FTC was DUSP5 knockdown dramatically induced cell proliferation, migration and invasion, and the effect was reversed after DUSP5 overexpression.

However, can DUSP5 serve as a prognostic molecular marker or pathogenic factor? To explore this point in greater detail, we analyzed the function of DUSP5 by in vitro experiment and found that downregulated DUSP5 induced an increase in proliferation and migration of FTC accompanied with overexpression of MMP9 and Vimentin and decreased E‐cadherin protein level, which were molecular markers of migration.^28^ On the contrary, upregulation of DUSP5 ameliorated the above damaging effect on the tumor growth and metastasis, which obtains preliminary evidence that DUSP5 acts as a key pathogenic factor to mediate the metastasis of FTC, not a biomarker.

In recent years, many studies have demonstrated that DUSP5 is widely involved in tumor progression and metastasis. Low DUSP5 expression was found in different types of tumors and can influence the prognosis of tumor patients by promoting tumor growth, invasion and metastasis.^29^, ^30^, ^31^ Yan *et al*. and Cai *et al*. both clarified that DUSP5 expression was negatively associated with advanced pathological stage in colorectal cancer and prostate cancer.^32^, ^33^ In pancreatic cancer cells, DUSP5 inhibited cell growth in a dose dependent manner.^34^ Nevertheless, the specific role of DUSP5 in the control of FTC metastasis which further contributes to poor prognosis has not yet been clearly elucidated.

It is worth noting that altered motility phenotypes are often associated with the tumor development and progression, and eventually metastases.^35^ Combined with the actuality that early distant metastasis frequently leads to worse prognosis of FTC, we may safely draw a conclusion that enhanced migration of tumor cells tends to be a potential behavior to aggravate the occurrence of worse prognosis of FTC.

Based on the distinct motility behavior between FTC and PTC, we explored the difference in prognosis and key factors which were involved in it between two DTCs. Our study identified a significant function of DUSP5 in the suppression of migration of FTC, even better than inhibition of proliferation, which strongly indicated that DUSP5 is a potential target for mediating early blood metastasis of FTC, subsequently leading to a poor prognosis of patients.

In summary, most clinicians today generally treat FTC and PTC equally and ignore the unique characteristics and severely poor outcome due to blood metastasis in FTC. We have clearly shown that DUSP5 inhibited the destructive behavior of FTC, especially for migration in vitro. In addition, a potential therapeutic target, DUSP5 that can mediate the early migration of FTC was investigated which provided the theoretical basis for early diagnosis and treatment of FTC. In future, additional studies will be necessary to explore the molecular regulation mechanism of DUSP5 in the early metastasis of FTC.

## Disclosure

The authors confirm that there are no conflicts of interest.

## Supporting information


**Figure S1** Kaplan‐Meier survival analysis was conducted to determine the associations between the DEGs (except DUSP5) expression levels and survival prognosis in cBioPortal.
