# Supplementary figures and images for: Integrated analysis identifies DUSP5 as a novel prognostic indicator for thyroid follicular carcinoma
Source: Thorac Cancer. 2019 Dec 10;11(2):336–45. doi: 10.1111/1759-7714.13270 (PMC6996982; doi:10.1111/1759-7714.13270)

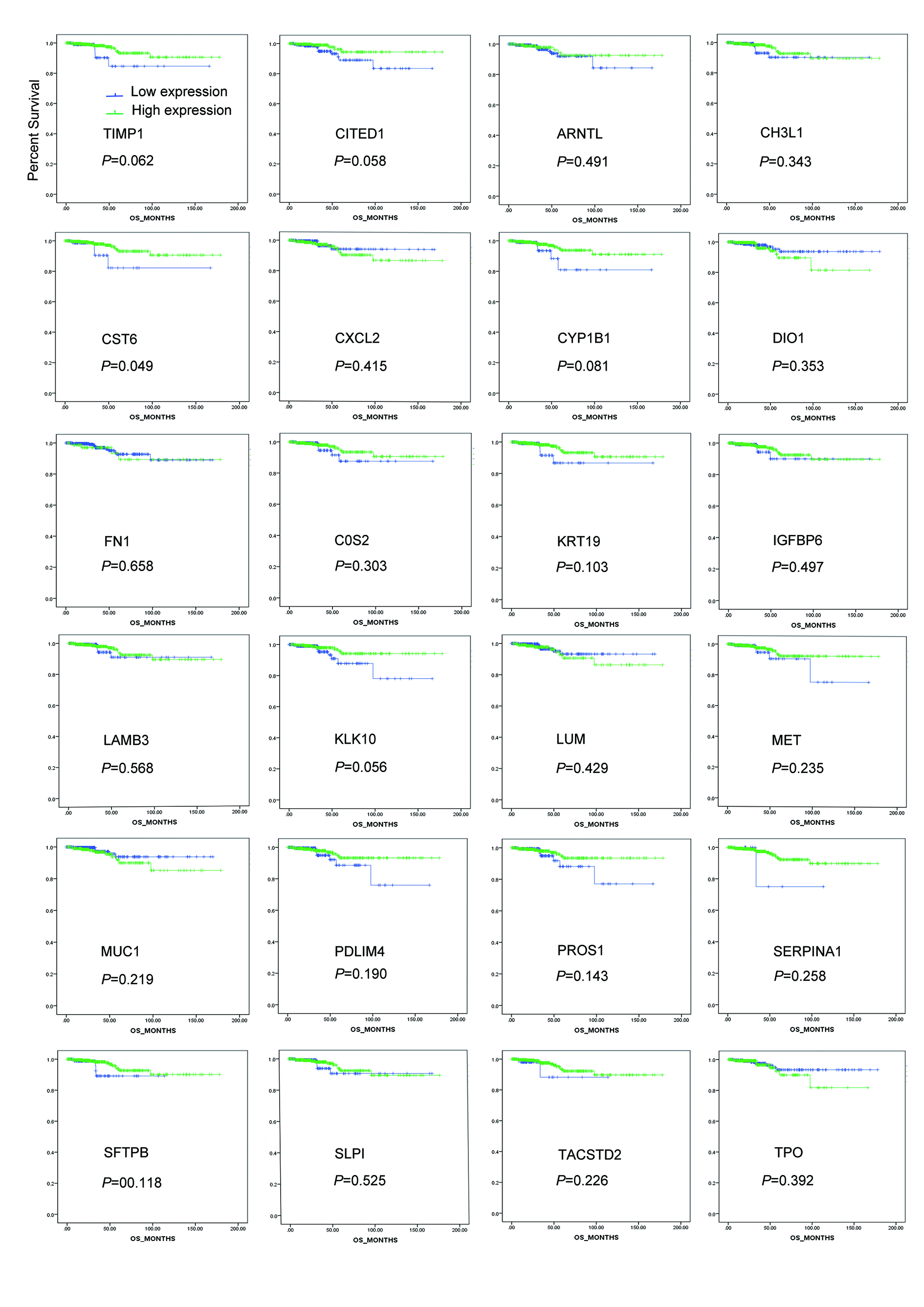

Supplement: Supplementary file 1 — Figure S1 Kaplan‐Meier survival analysis was conducted to determine the associations between the DEGs (except DUSP5) expression levels and survival prognosis in cBioPortal. [file TCA-11-336-s001.tif]
